# Spatial and temporal variations in airborne *Ambrosia* pollen in Europe

**DOI:** 10.1007/s10453-016-9463-1

**Published:** 2016-11-17

**Authors:** B. Sikoparija, C. A. Skjøth, S. Celenk, C. Testoni, T. Abramidze, K. Alm Kübler, J. Belmonte, U. Berger, M. Bonini, A. Charalampopoulos, A. Damialis, B. Clot, Å. Dahl, L. A. de Weger, R. Gehrig, M. Hendrickx, L. Hoebeke, N. Ianovici, A. Kofol Seliger, D. Magyar, G. Mányoki, S. Milkovska, D. Myszkowska, A. Páldy, C. H. Pashley, K. Rasmussen, O. Ritenberga, V. Rodinkova, O. Rybníček, V. Shalaboda, I. Šaulienė, J. Ščevková, B. Stjepanović, M. Thibaudon, C. Verstraeten, D. Vokou, R. Yankova, M. Smith

**Affiliations:** 1 BioSense Institute - Research Institute for Information Technologies in Biosystems, University of Novi Sad, Novi Sad, Serbia; 2 National Pollen and Aerobiology Unit, Institute of Science and the Environment, University of Worcester, Henwick Grove, Worcester, WR2 6AJ UK; 3 Biology Department, Science Faculty, Uludağ University, Bursa, Turkey; 4 Local Health Authority Milano Città Metropolitana, Milan, Italy; 5 Center of Allergy and Immunology, Tbilisi, Georgia; 6 Swedish Museum of Natural History, Stockholm, Sweden; 7 Institute of Environmental Science and Technology (ICTA), Universitat Autònoma de Barcelona, Bellaterra, Barcelona, Spain; 8 Departament de Biologia Animal, Biologia Vegetal i Ecologia, Universitat Autònoma de Barcelona, Bellaterra, Barcelona, Spain; 9 Department of Oto-Rhino-Laryngology, Medical University of Vienna, Vienna, Austria; 10 Department of Ecology, School of Biology, Aristotle University of Thessaloniki, Thessaloniki, Greece; 11 Chair and Institute of Environmental Medicine, UNIKA-T, Technical University of Munich and Helmholtz Zentrum München - German Research Center for Environmental Health, Augsburg, Germany; 12 Federal Office of Meteorology and Climatology MeteoSwiss, Zurich, Switzerland; 13 Department of Plant and Environmental Sciences, University of Gothenburg, Gothenburg, Sweden; 14 Department of Pulmonology, Leiden University Medical Center, Leiden, The Netherlands; 15 Belgian Aerobiology Network, Scientific Institute of Public Health, Brussels, Belgium; 16 Faculty of Chemistry-Biology-Geography, West University of Timisoara, Timisoara, Romania; 17 Institute of Public Health of the Republic of Slovenia, Ljubljana, Slovenia; 18 National Public Health Center, Budapest, Hungary; 19 Institute of Occupational Health - WHO Collaborating Center, Skopje, Republic of Macedonia; 20 Department of Clinical and Environmental Allergology, Jagiellonian University Medical College, Kraków, Poland; 21 Institute for Lung Health, Department of Infection, Immunity & Inflammation, University of Leicester, Leicester, UK; 22 Astma-Allergi Danmark, Roskilde, Denmark; 23 Faculty of Geography and Earth Sciences, University of Latvia, Riga, Latvia; 24 Vinnitsa National Pirogov Memorial Medical University, Vinnitsa, Ukraine; 25 Faculty of Medicine, Masaryk University, Brno, Czech Republic; 26 V. F. Kuprevich Institute for Experimental Botany of the NAS of Belarus, Minsk, Belarus; 27 Department of Environmental Research, Siauliai University, Šiauliai, Lithuania; 28 Faculty of Natural Sciences, Comenius University Bratislava, Bratislava, Slovakia; 29 Institute of Public Health “Dr Andrija Štampar”, Zagreb, Croatia; 30 Réseau National de Surveillance Aérobiologique (R.N.S.A.), Brussieu, France; 31 Clinical Center of Allergology, University Hospital Sofia, Sofia, Bulgaria; 32 Institute of Science and the Environment, University of Worcester, Henwick Grove, Worcester, WR2 6AJ UK

**Keywords:** Aerobiology, Ragweed, Invasive alien species, Allergen, Exposure

## Abstract

The European Commission Cooperation in Science and Technology (COST) Action FA1203 “SMARTER” aims to make recommendations for the sustainable management of *Ambrosia* across Europe and for monitoring its efficiency and cost-effectiveness. The goal of the present study is to provide a baseline for spatial and temporal variations in airborne *Ambrosia* pollen in Europe that can be used for the management and evaluation of this noxious plant. The study covers the full range of *Ambrosia artemisiifolia* L. distribution over Europe (39°N–60°N; 2°W–45°E). Airborne *Ambrosia* pollen data for the principal flowering period of *Ambrosia* (August–September) recorded during a 10-year period (2004–2013) were obtained from 242 monitoring sites. The mean sum of daily average airborne *Ambrosia* pollen and the number of days that *Ambrosia* pollen was recorded in the air were analysed. The mean and standard deviation (SD) were calculated regardless of the number of years included in the study period, while trends are based on those time series with 8 or more years of data. Trends were considered significant at *p* < 0.05. There were few significant trends in the magnitude and frequency of atmospheric *Ambrosia* pollen (only 8% for the mean sum of daily average *Ambrosia* pollen concentrations and 14% for the mean number of days *Ambrosia* pollen were recorded in the air). The direction of any trends varied locally and reflected changes in sources of the pollen, either in size or in distance from the monitoring station. Pollen monitoring is important for providing an early warning of the expansion of this invasive and noxious plant.

**Electronic supplementary material:**

The online version of this article (doi:10.1007/s10453-016-9463-1) contains supplementary material, which is available to authorized users.

## Introduction

*Ambrosia artemisiifolia* L. (common or short ragweed) has been considered to be an invasive and alien plant by the European and Mediterranean Plant Protection Organization since 2004 (Brunel et al. 2010). It is an important weed in agriculture and source of highly allergenic pollen. The plant has now become naturalized in Europe and frequently forms part of the flora (Smith et al. 2013). The prevalence of sensitization to *Ambrosia* pollen allergens is increasing in Europe and reflects the expansion of *Ambrosia* populations (Burbach et al. 2009b).

Aerobiological monitoring sites routinely collect and report levels of atmospheric pollen across Europe. The samples are examined by light microscopy, and the data can be used for a variety of purposes, including being an early warning of the spread of invasive, wind-pollinated (anemophilous) plants like *Ambrosia artemisiifolia.* The pollen grains of *A. artemisiifolia* are morphologically similar to the other introduced species of *Ambrosia* in Europe, *A. trifida* L., *A. tenuifolia* Spreng. and *A. psilostachya* DC. (=*A. coronopifolia* Torr. & Gray) as well as the native *A. maritima* L. (Smith et al. (2013) and references therein). As a result, the pollen grains of *Ambrosia* species are identified to genus level by monitoring stations.

The threat posed by *Ambrosia* has been identified, and efforts to reduce the negative impacts on the human population have started to be implemented at national and European levels (Smith et al. 2013). The European Commission Cooperation in Science and Technology (COST) Action FA1203 “SMARTER” (http://ragweed.eu) aims to make recommendations for the sustainable management of *Ambrosia* across Europe and for monitoring its efficiency and cost-effectiveness. This study has been conducted within the frame of Working Group 4 of SMARTER, with the goal of providing a baseline for spatial and temporal variations in airborne *Ambrosia* pollen in Europe that can be used for the management and evaluation of this noxious plant.

## Materials and methods

### Collection of pollen data

Pollen data were collected by airborne pollen-monitoring networks across Europe by using volumetric spore traps of the Hirst design (Hirst 1952), thereby ensuring the comparability of the data, and samples were analysed using methods recommended in the literature (Galán et al. 2014). Daily average pollen concentrations are expressed as particles per cubic metre of air (P m^−3^) (Comtois 1998). The protocol for collating the pollen data was based on the methods described by Thackeray et al. (2010) and Ziello et al. (2012) that were adjusted for the needs of this study. Datasets were restricted to the period August–September, which is the principal flowering period of *Ambrosia* (Bonini et al. 2015), and the years 2004–2013 only. Within this period, the analyses examined the sum of daily average *Ambrosia* pollen concentrations and the number of days when daily average concentrations exceeded 1 P m^−3^. The study focuses on a 10-year period (2004–2013). This is because it allows comparison between datasets, as not all sites have been monitoring airborne pollen for long periods of time (i.e. >10 years).

**Fig. 1:**
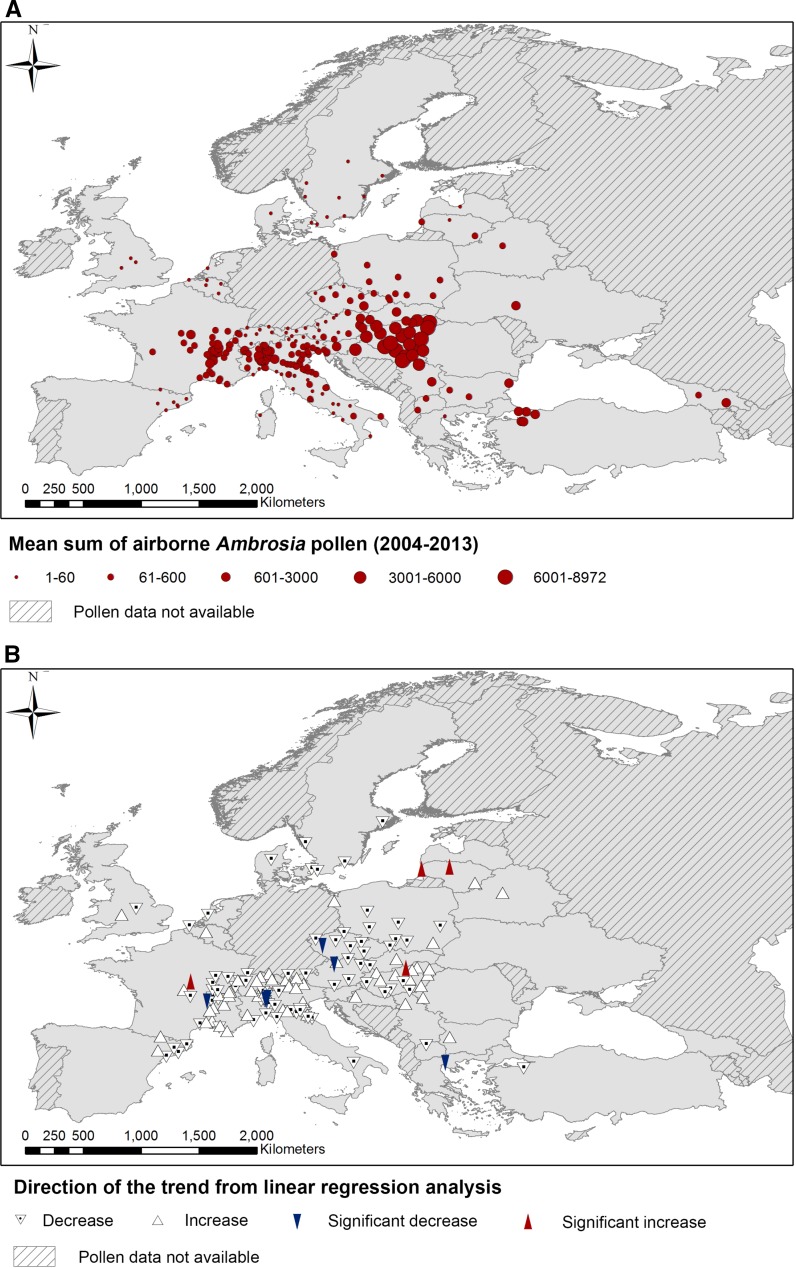
The location of pollen-monitoring sites involved in the study, showing the following results calculated using the mean sum of available daily airborne pollen concentrations from August to September during the years 2004–2013: **a** the mean sums of airborne *Ambrosia* pollen concentration; **b** significant trends and the direction of slope of linear regression analysis (only sites with ≥8 years of pollen data). Regions where pollen data were not available are also depicted

**Fig. 2:**
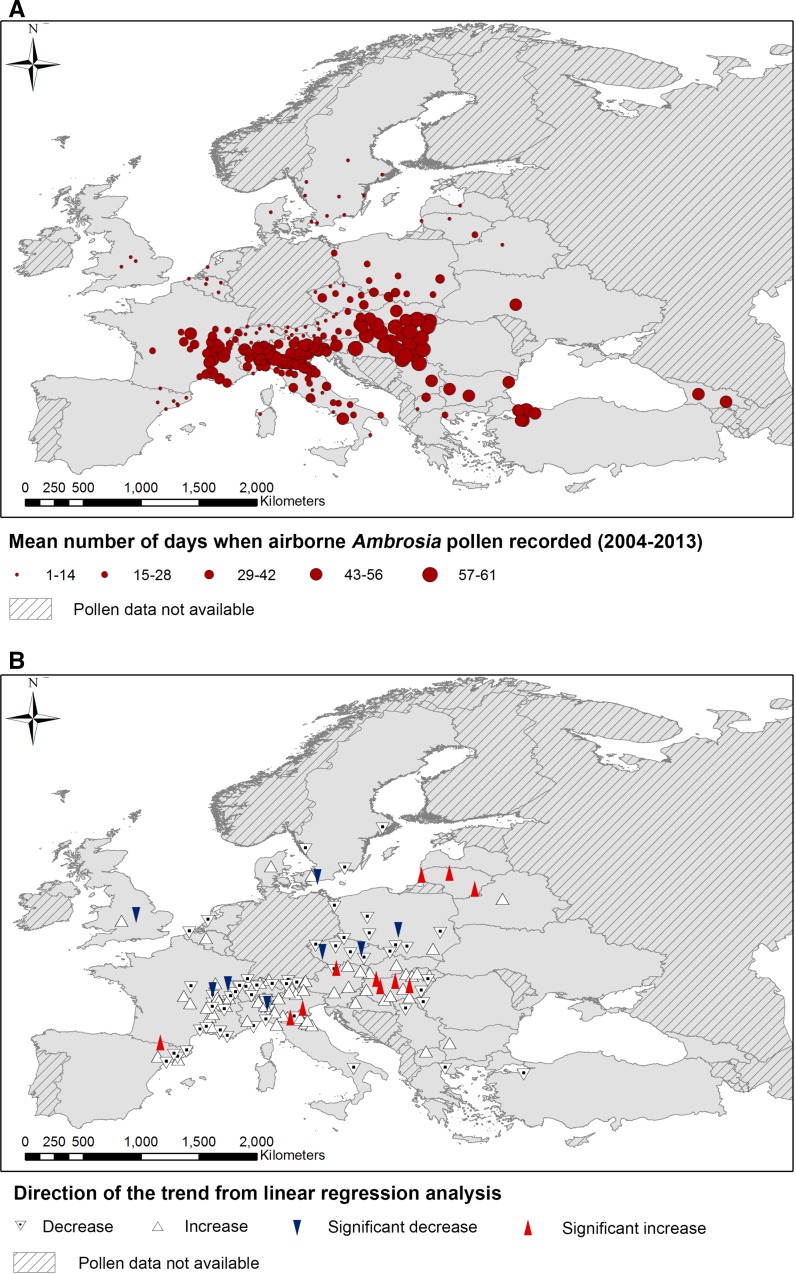
The location of pollen-monitoring sites involved in the study, showing the following results calculated using the available number of days when airborne *Ambrosia* pollen data were recorded during August–September for the years 2004–2013: **a** the mean number of days when *Ambrosia* pollen was recorded (maximum 61); **b** significant trends and the direction of slope of linear regression analysis (only sites with ≥8 years of pollen data). Regions where pollen data were not available are also depicted

Participants included individual sites as well as regional and national pollen-monitoring networks, encompassing a number of countries involved in the COST SMARTER network. *Ambrosia* pollen data were unavailable for the study from several countries (Figs. 1, 2). For instance, due to constraints in time and resources, a number of sites routinely cease monitoring in August or September, while *Ambrosia* plants are flowering. At other sites, such as in Portugal, *Ambrosia* pollen is rarely found in the atmosphere and as such it is not considered to be of allergological importance. As a result, *Ambrosia* pollen grains are only identified to family level (i.e. Asteraceae) (E. Caeiro, personal communication). This also applies to parts of Spain, but Catalonia was included because *Ambrosia* pollen is recorded in this region (Fernández-Llamazares et al. 2012). The German Pollen Information Service (PID) did not participate in the current study, but data about spatial variations in ragweed populations and the annual *Ambrosia* pollen index in Germany (2012–2014) are available in the recent paper by Buters et al. (2015). It is also important to note that for several spatially large countries (i.e. Romania, Turkey and Ukraine) pollen-monitoring networks are not dispersed over the entire territory, and so the data included in this study are not representative of the entire area of these vast countries.

### Data preprocessing and statistical analysis

Datasets were examined for missing values and irregularities. Years that had more than 7 days missing from the flowering period of *Ambrosia* were removed from the analysis because it was deemed that this would have a noticeable effect on the results. The mean and standard deviation (SD) were calculated regardless of the number of years included in the study period. On the other hand, linear trends in the sum of pollen recorded annually and the number of days when *Ambrosia* pollen grains were recorded during August and September each year were calculated (Thackeray et al. 2010; Ziello et al. 2012; Smith et al. 2014) for sites with at least 80% records (≥8 years) in the study period and the following results presented: slope of the simple linear regression over time, standard error of the regression slope (SE), probability level (*p*) and coefficient of determination (*R* ^2^). Trends were considered significant with probability levels <0.05.

## Results and discussion

A total of 1730 datasets (years of pollen data), from 242 locations, were included in the analysis. Trends were calculated for 143 locations (see Appendix S1 in Supporting Information). Trends were only calculated for sites with ≥8 years of pollen data, but mean values for the sum of daily average *Ambrosia* pollen concentrations recorded annually and the number of days that *Ambrosia* pollen was recorded in the air were included for all sites, regardless of the length of the dataset, because it allowed valuable data to be included in the analysis. For example, aerobiological monitoring in Georgia is still developing and only 2 years of data were available for this study. However, habitat suitability analysis for the country predicts that *Ambrosia artemisiifolia* L. has a distribution of 24%, increasing to 40% over the next 50 years (Thalmann et al. 2015), which is reflected in this study by the atmospheric pollen levels and number of days in August and September that *Ambrosia* pollen is recorded (Figs. 1a, 2a).

### The sum of pollen recorded annually

This study agrees with previous work showing that France, Northern Italy, the Pannonian Plain and Ukraine (Fig. 1a) record some of the highest levels of airborne *Ambrosia* pollen in Europe (Skjøth et al. 2013). Mean levels of atmospheric *Ambrosia* pollen tend to decrease away from these centres, e.g. towards the Atlantic and Baltic coasts in the north and the Mediterranean in the south, although elevated levels of atmospheric *Ambrosia* pollen were also recorded in the Black Sea region in Turkey and Georgia. It has been hypothesized that airborne *Ambrosia* pollen in Turkey originates from both local sources and long-distance transport (Zemmer et al. 2012). The plant had previously been reported to be well established in Northeast Anatolia (Byfield and Baytop 1998), and experts belonging to COST SMARTER recently confirmed the occurrence of limited populations of *Ambrosia* in the vicinity of Samsun near to the Black Sea coast (B. Chauvel, personal communication). It is also predicted that Georgia has notable local *Ambrosia* populations (Thalmann et al. 2015).

Out of a total of 143 trends for the sum of atmospheric *Ambrosia* pollen calculated using 8 or more years of data, only 11 were significant (8%) and 7 of these were towards significant decreases in the amount of airborne *Ambrosia* pollen. Several of these significant decreases were calculated at sites already considered to be centres of *Ambrosia* infestation, i.e. Rhône-Alpes region in France and Northern Italy. Such decreases may be the result of successful control measures against *Ambrosia* in these areas or, in the case of Northern Italy, the accidental introduction of the *Ophraella communa* leaf beetle that has coincided with a significant decrease in atmospheric concentrations of *Ambrosia* pollen in the region (Bonini et al. 2015, 2016). This confirms that factors determining the rate of spread of *Ambrosia* within its current climatic niche (Hamaoui-Laguel et al. 2015) can affect pollen concentrations even without changing the plant distribution.

Hamaoui-Laguel et al. (2015) predicted that atmospheric *Ambrosia* pollen concentrations would increase up to a factor of two in current high pollen level areas like the Pannonian Plain but, as yet, this has not been seen. Instead, this study has shown that significant increases in the amount of airborne *Ambrosia* pollen tended to be in areas considered to be at the forefront of *Ambrosia* expansion, such as Nevers in France and Salgótarján in Hungary, which are on the periphery of the main centres of *Ambrosia* (Skjøth et al. 2010; Thibaudon et al. 2014; Karrer et al. 2015). Although significant increases were also witnessed at sites situated some distance away from areas traditionally considered to be the heart of *Ambrosia* infestation, i.e. Lithuania. This discrepancy between monitored pollen data and values expected by the models implies that future distribution of invasive weeds, including their abundance, is affected both by climate conditions and by local anthropogenic influences. Therefore, assessment of *Ambrosia* biogeography and rates of distribution change in the plant’s non-native distribution range, would benefit from integrating population dynamics and anthropogenic drivers, such as mechanisms for local and long-distance seed dispersal (Chapman et al. 2016).

### The number of days when Ambrosia pollen was recorded

The number of days when *Ambrosia* pollen grains were recorded during August and September generally decreased with increased distance away from known centres of *Ambrosia* infestation (Fig. 2a). This probably reflects the intermittent nature of atmospheric transport episodes from areas with notable sources of *Ambrosia* pollen to areas where the plant is less common, or not found at all (Stach et al. 2007; Smith et al. 2008; Sikoparija et al. 2009; Kasprzyk et al. 2011; Šikoparija et al. 2013; Sommer et al. 2015). The results show that *Ambrosia* pollen is also frequently recorded in the Balkans, Greece, Turkey and Georgia. Some of these countries record lower *Ambrosia* pollen levels (Fig. 1a), but the number of days that people are exposed to this aeroallergen is high. Frequent exposure to low amounts of *Ambrosia* pollen allergens might explain why the crude clinically relevant sensitization rate to *Ambrosia* pollen allergens presented by Burbach et al. (2009a) for Greece (~5%), is similar to those recorded in Austria (Vienna), France (Montpellier) and Poland (Lodz) that are closer to the *Ambrosia* heartlands.

The amount of significant trends in the number of days when *Ambrosia* pollen was recorded was low (just 14%). As with the sum of airborne *Ambrosia* pollen recorded in August–September, there is a tendency for sites removed from centres of *Ambrosia* distribution to exhibit significant trends towards more days with *Ambrosia* pollen in the air (Fig. 2a). Where local sources of *Ambrosia* plants are not present or only sporadically recorded (e.g. Lithuania, Sauliene et al. 2011), these trends towards more frequent exposure to airborne *Ambrosia* pollen might reflect: (1) increases in the magnitude of nearby sources, (2) a decrease in the distance between the site and the source caused by successful invasions (Leiblein-Wild et al. 2014) that would increase the risk of pollen being transported to an area more frequently or (3) conditions becoming more conducive for atmospheric transport (Hamaoui-Laguel et al. 2015).

Prior to conducting this study, it was expected that *Ambrosia* pollen levels would be comparatively stable in areas considered to be centres of *Ambrosia* distribution where the plant has been present for considerable lengths of time. If anything, it was anticipated that the amount of airborne *Ambrosia* pollen or the frequency that *Ambrosia* pollen was recorded in the air might decrease in these areas due to factors such as management. However, significant increases in the number of days with *Ambrosia* pollen in the air were witnessed in Hungary on the Pannonian Plain where the plant has been considered to be a problem weed since the 1960s (Smith et al. (2013) and references therein), which suggests that episodes of airborne *Ambrosia* pollen are actually increasing in frequency in some areas. This concurs with Hamaoui-Laguel et al. (2015) who postulate that conditions will become more favourable for the release and atmospheric accumulation of *Ambrosia* pollen from the plant. Nevertheless, it is possible that, for sites with stable *Ambrosia* populations that record *Ambrosia* pollen almost every day during August and September, missing days will have an impact on the results. For this reason, care was taken to ensure that years with more than 7 missing values (e.g. due to trap failure) during the principal flowering period of *Ambrosia* were not included in the analysis.

There are a number of other local differences in the number of days that *Ambrosia* pollen has been recorded in different regions (Fig. 2b). For instance, *Ambrosia* plants are rarely recorded in Lithuania to the north-east of the study area (Sauliene et al. 2011). The number of *Ambrosia* plants in Lithuania is not increasing, but an increase in atmospheric concentrations of *Ambrosia* pollen has been noted and linked with the occurrence of south-easterly winds and potential long-distance transport (Šaulienė and Veriankaitė 2012). Long-distance transport of *Ambrosia* pollen has also been witnessed in Catalonia to the south-west of the study area, although airborne pollen records in the region could also be substantially influenced by local populations of *Ambrosia* species that reportedly increased by 324% (Fernández-Llamazares et al. 2012). This might explain the significant increase in the number of days when *Ambrosia* pollen has been recorded in the air of north-eastern Spain in this study. In Northern Italy, on the other hand, a significant decrease in the number of days with airborne *Ambrosia* pollen was witnessed to the North of Milan (Vertemate con Minoprio). This is the region where the oligophagous leaf beetle *Ophraella communa*, which is known to feed on *A. artemisiifolia*, has been sighted (Müller-Schärer et al. 2014) and potentially linked to decreases in atmospheric concentrations of *Ambrosia* pollen reported at some sites (Bonini et al. 2015). Conversely, significant increases in the number of days with airborne *Ambrosia* pollen were seen at sites situated in the Po Valley to the south (Modena) and east (Padua), which might indicate further expansion of *Ambrosia* in these areas.

This study shows spatial and temporal variations in the magnitude of airborne *Ambrosia* pollen concentrations and the number of days that the pollen is recorded in the air during the principal flowering period of *Ambrosia* over a 10-year period (2004–2013) at sites across Europe. The map of *Ambrosia* distribution in Europe is constantly changing with the inclusion of new data, as seen with the addition of Georgia in this study. The number of significant trends in the magnitude and frequency of atmospheric *Ambrosia* pollen is low (only 8% for the mean sum of daily average *Ambrosia* pollen concentrations and 14% for the mean number of days *Ambrosia* pollen were recorded in the air), and the direction of any changes varies locally. These trends reflect variations in sources of the pollen. Significant decreases can be related to external factors such as the introduction of control measures or herbivores that target the plant. Significant increases, on the other hand, can relate to expansions in the size of the source or a shortening of the distance from the source to the monitoring station, thereby increasing the magnitude and frequency of atmospheric pollen concentrations. However, the influence of short-term variations in local weather conditions or long-term effects climate change on the production, release and dispersion of *Ambrosia* pollen cannot be discounted. This study highlights the importance of pollen-monitoring networks, especially those that do not currently record *Ambrosia,* to commence actively looking for the pollen of this invasive and noxious plant, even if it is not currently considered to be an important aeroallergen in certain regions. This will provide an early warning of its expansion to new areas.

## Electronic supplementary material

Below is the link to the electronic supplementary material.

Mean, Standard Deviation and Linear trends calculated for sites included in the study (XLSX 325 kb)
